# Carbapenem-Resistant *Enterobacterales* in the Western Balkans: Addressing Gaps in European AMR Surveillance Map

**DOI:** 10.3390/antibiotics13090895

**Published:** 2024-09-19

**Authors:** Snezana Brkic, Ivana Cirkovic

**Affiliations:** 1 Institute for Laboratory Diagnostics “Konzilijum”, 11000 Belgrade, Serbia; 2 Institute of Microbiology and Immunology, Faculty of Medicine, University of Belgrade, 11000 Belgrade, Serbia; ivana.cirkovic@med.bg.ac.rs

**Keywords:** Western Balkans (WBs), antimicrobial resistance (AMR) data, carbapenem-resistant *Enterobacterales* (CRE)

## Abstract

In the context of global efforts to combat antimicrobial resistance (AMR), the importance of comprehensive AMR data is more crucial than ever. AMR surveillance networks, such as the European Antimicrobial Resistance Surveillance Network (EARS-Net) and the Central Asian and European Surveillance of Antimicrobial Resistance (CAESAR), support member states in obtaining high-quality AMR data. Nevertheless, data gaps persist in some countries, including those in the Western Balkans (WBs), a region with high AMR rates. This review analyzed existing research on carbapenem-resistant *Enterobacterales* (CRE) to better understand the AMR landscape in the WB countries. The most prevalent CRE was *Klebsiella pneumoniae*, followed by *Escherichia coli*, *Enterobacter cloacae*, and *Proteus mirabilis*, with sporadic cases of *Morganella morganii*, *Providencia* spp., *Klebsiella oxytoca*, and *Citrobacter sedlakii*. Carbapenemase production was identified as the most common mechanism of carbapenem resistance, but other resistance mechanisms were not investigated. An increasing trend in carbapenem resistance has been observed over the last decade, alongside a shift in carbapenemase epidemiology from the NDM type in 2013–2014 to the OXA-48 type in recent years. Few studies have applied whole-genome sequencing for CRE analysis, which has demonstrated the spread of resistance determinants across different niches and over time, emphasizing the importance of molecular-based research. The overall low number of studies in the WB countries can be attributed to limited resources, highlighting the need for enhanced support in education, training, technology, and equipment to improve data collection and evaluation.

## 1. Introduction

A dramatic increase in antimicrobial resistance (AMR) in recent decades, followed by a rapid spread of resistant clones at the global level, represents one of the main challenges of modern medicine. The most important aspect of AMR is the rising trend in deaths associated with it. Drug-resistant bacteria are responsible for more than 700,000 deaths each year, but this concerning number might be underestimated due to poor reporting and surveillance [1]. To tackle this problem, the World Health Organization (WHO) presented its Global Action Plan (GAP) on AMR ten years ago [2]. The GAP proposed multiple actions at the global and national level, and one of its main objectives is encouraging investments in the development of new antimicrobials [2]. To support this objective, the WHO developed the Bacterial Priority Pathogen List (BPPL) of public health importance to guide research, development, and strategies to prevent and control AMR. The first BPPL from 2017 was updated in 2024 and includes 15 families of antibiotic-resistant pathogens grouped into critical, high, and medium categories of priority. Among critical priority pathogens are carbapenem-resistant *Acinetobacter baumannii*, third-generation cephalosporin-resistant and carbapenem-resistant *Enterobacterales*, and rifampicin-resistant *Mycobacterium tuberculosis* [3].

The WHO critical priority pathogens are among the top six species each responsible for more than 250,000 deaths attributable to and associated with bacterial AMR in 2019 [4]. Infections with carbapenem-resistant *Enterobacterales* (CRE) are linked to a greater risk of death and an increasing effect on mortality over time, which may reflect the difficulty of the CRE infection treatment [5]. Moreover, the pathogen–drug combinations with the most resistance-attributable deaths in 2019 were the third-generation cephalosporin-resistant *Escherichia coli*, carbapenem-resistant *A. baumannii*, fluoroquinolone-resistant *E. coli*, carbapenem-resistant *Klebsiella pneumoniae*, and third-generation cephalosporin-resistant *K. pneumoniae* [4].

**Figure 1:**
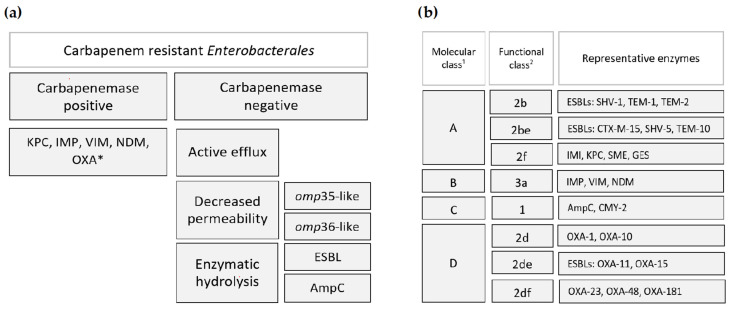
(**a**) Mechanisms of carbapenem resistance in *Enterobacterales*. * The “Big Five” and (**b**) classification of β-lactamases. ^1^ Ambler’s classification and ^2^ Bush–Jacoby’s classification.

It is obvious that the backbone of the problem is resistance to carbapenems, “the last resort” therapy for multidrug-resistant strains, especially third-generation cephalosporin-resistant *E. coli* and *K. pneumoniae*. The most important mechanism of carbapenem resistance in *Enterobacterales* is hydrolysis by carbapenemase enzymes. In carbapenemase-negative strains, there are several different mechanisms of resistance (Figure 1a). Mutations in porins on the bacterial surface, e.g., Omp35-like and Omp36-like, compromise the diffusion of carbapenems into the cell. This enables other enzymes, especially extended-spectrum β-lactamases (ESBLs) and AmpC enzymes, to hydrolyze the antibiotics [6,7]. Increased efflux activity, which manifests as overexpression of the efflux pumps, contributes significantly to overall resistance to carbapenems [6]. These non-carbapenemase-related mechanisms were dominant in some countries at the beginning of the 21st century [6], but the global trend in increase in CRE prevalence is related to production of carbapenemases. The exceptional diversity of β-lactamases, including carbapenemases, could be perceived through two main classifications of β-lactamases, namely Ambler’s molecular classes and Bush–Jacoby’s functional classification (Figure 1b) [7,8].

The detection of the first plasmid-encoded carbapenemase in *Serratia marcescens* in Japan during the early 1990s marked a critical moment in the global epidemiology of carbapenem resistance in *Enterobacterales* [9]. The spread of resistance was inevitable due to the diversity of mobile genetic elements carrying carbapenemase genes, enabling the transfer of resistance between *Enterobacterales* of the same species, as well as of different species [10,11]. Numerous epidemiological studies have investigated the global distribution of carbapenemases, identifying the so-called “Big Five” (KPC, IMP, VIM, NDM, and OXA-48) as the most clinically important types worldwide [12].

KPC (*Klebsiella pneumoniae* carbapenemase) is considered to be the most widespread type globally [13]. It was first detected in the United States in 1996, where it is still the most prevalent type among *Enterobacterales*. KPC belongs to Ambler’s class A, as a serin-carbapenemase, and to the 2f functional group. This enzyme hydrolyses all β-lactam antibiotics and it is not inhibited by β–lactamase inhibitors [7]. Highly conserved transposon Tn*4401* is the main carrier of the *bla*_KPC_ gene, usually associated on the same plasmid with other resistance genes, like ESBL, aminoglycoside, and fluoroquinolone resistance genes [12,14]. It is obvious that KPC-producing strains represent a great challenge both for therapy and for infection prevention and control.

In Ambler’s class B and Bush–Jacoby’s 3a group are IMP (imipenem-resistant), VIM (Verona integron-encoded metallo-β-lactamase) and NDM (New Delhi metallo-β-lactamase). These carbapenemases are metallo-β-lactamases (MBLs), zinc-dependent enzymes with hydrolytic activity to all β-lactams, except monobactams [12]. This structural characteristic also results in a resistance to β-lactamase inhibitors in clinical use (clavulanic acid, tazobactam, avibactam), but metal chelating agents such as ethylenediaminetetraacetic acid (EDTA) could inhibit MBLs in vitro [15]. IMP was the first plasmid-encoded carbapenemase detected among *Enterobacterales*, found in an isolate of *S. marcescens* in Japan in 1991 [9]. It is not as widespread as other types but is endemic in the region of East Asia [10,12]. VIM carbapenemase was first identified in *Pseudomonas aeruginosa* in 1996 and was less frequently associated with resistance to carbapenems in *Enterobacterales* than other MBLs [10]. Several years later, in the early 2000s, VIM-producing *K. pneumoniae* became endemic in Southern Europe and South Asia [10,11,12]. In contrast to IMP and VIM, NDM is the most frequent and the most widely spread MBL in *Enterobacterales*. NDM was first detected in Sweden in 2008, in a *K. pneumoniae* isolate from a patient linked to India [16]. Beside India, NDM is endemic in Balkan countries, Northern Africa, and the Arabian Peninsula [10,12]. Numerous studies observed a high diversity of *bla*_NDM_ genomic background, not related to any specific clone, species, or plasmid type, which explains a global dissemination of this carbapenemase [10,12]. An additional aspect of successful NDM spreading is the detection of *bla*_NDM_ genes in different environmental sources, which has a significant impact on the effectiveness of AMR control measures [11,14].

OXA (Oxacillinase) belongs to Ambler’s class D and functional group 2df. This type of carbapenemase has a weak hydrolytic activity to carbapenems, but as they are nearly always associated with other resistance mechanisms, OXA-positive isolates are usually phenotypically resistant to carbapenems [10]. This is the most heterogeneous group of carbapenemases, with more than 750 variants [17]. The most important type in *Enterobacterales* is OXA-48, first described in an isolate of *K. pneumoniae* from Turkey in 2003 [12]. Since then, it has been disseminated very successfully from its endemic region in Turkey, the Middle East countries (Lebanon, Jordan, Oman, Iran, and Saudi Arabia), and North Africa (Morocco, Algeria, Tunisia, and Egypt) to all continents except Antarctica [12,14,17]. This global expansion of OXA-48-positive clones is related, in contrast to NDM, to a particular genetic entity—Tn*1999* on IncL/M self-conjugative pOXA-48a-like plasmid [12,17].

All aforementioned mechanisms of resistance clearly suggest that treating infections caused by CRE has become very difficult [10]. One of the few antibiotics that remain active against CRE is polymyxin E or colistin. Regardless of the side effects, such as nephrotoxicity and neurotoxicity, colistin re-emerged as a treatment option for CRE [18]. Thereafter, its increased clinical use led to an increased resistance rate, both chromosomally mediated and a more concerning plasmid-mediated resistance [18,19]. In *Enterobacterales*, acquired resistance to colistin is mainly associated with mutations in chromosomal genes. These include regulatory genes, such as PmrAB and PhoPQ two-component systems or the *mgr*B gene, and lead to enzymatic modifications of the binding site for colistin—a lipid A in a lipopolysaccharide of the cell wall [18,19]. Chromosomally mediated resistance is transferred vertically, and its dissemination is related to the spread of colistin-resistant CRE clones, e.g., *K. pneumoniae* ST258 [18]. The detection of plasmid-mediated colistin resistance in *Enterobacterales* involved an *mcr*-1 gene in an isolate of *E. coli* in China in 2015 [20], which alarmed public health institutions all over the world. Since then, numerous variants of *mcr-1* to *mcr-10* genes have been described in different species. What is more concerning is the heterogeneity of the origin of *mcr* genes, as they are detected in humans, animals, plants, water, and other environmental sources, implicating a tremendous effort needed to control the spread of resistance [16,19,21].

## 2. AMR Trends in the Western Balkans

**Figure 2:**
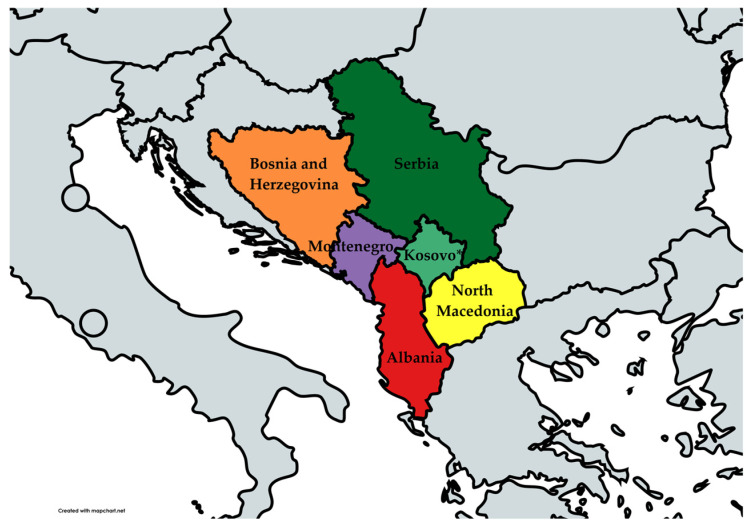
The Western Balkan countries.

The Western Balkans (WBs) consists of Albania, Bosnia and Herzegovina, North Macedonia, Montenegro, Kosovo *, and Serbia (* this designation is without prejudice to positions on status and is in line with UNSCR 1244 and the ICJ Opinion on the Kosovo Declaration of Independence) [22]. All have a perspective to accede to the European Union and hence are also called “enlargement countries” (Figure 2). As the systematic and continuous surveillance of AMR trends is one of the key segments of tackling the AMR problem, and since resistant bacteria spread beyond political boundaries, it is of utmost importance to have comprehensive AMR data for the region of the WB countries as well.

Back in 2013, the European Centre for Disease Prevention and Control (ECDC) was funding a project on carbapenemase-producing bacteria in Europe (EuSCAPE) [23]. Among 38 participating countries included in a survey of carbapenemase-producing *Enterobacteriaceae* (CPE) in Europe were all the WB countries. The results were categorized into the following seven stages that describe the national spread of CPE: 0, 1, 2a, 2b, 3, 4, and 5, from no cases (stage 0) to an endemic situation (stage 5) [23]. The results for the WB countries were diverse. In Montenegro and North Macedonia, no CPE were detected, while in Serbia and Bosnia and Herzegovina, there was a sporadic occurrence of CPE (stage 1). Albania was at stage 2a with a single hospital outbreak, and sporadic hospital outbreaks were reported in Kosovo (stage 2b) [23]. The survey was repeated in 2018, and the results showed a worsening of the epidemiological situation for most WB countries. In Serbia, there was a significant increase in CPE spread, which was categorized as epidemiological stage 4 with an inter-regional spread of CPE [24]. In Bosnia and Herzegovina, sporadic hospital outbreaks were reported (stage 2b), while in North Macedonia, a single hospital outbreak (stage 2a) was registered. In Montenegro, the sporadic occurrence of CPE (stage 1) was described, as well as in Albania and Kosovo [24].

Importantly, the European Antimicrobial Resistance Surveillance Network (EARS-Net), which includes 26 European Union (EU)/European Economic Area (EEA) countries, together with the Central Asian and European Surveillance of Antimicrobial Resistance (CAESAR) network, which supports 21 more European countries from the WHO European Region (excluding EU/EEA), perform surveillance and release annual reports of AMR trends [25]. Joint reports of these networks include data on invasive bacterial isolates (isolates from blood and cerebrospinal fluid) of eight bacterial pathogens commonly causing infections in humans, namely *E. coli*, *K. pneumoniae*, *P. aeruginosa*, *Acinetobacter* species, *Streptococcus pneumoniae*, *Staphylococcus aureus*, *Enterococcus faecalis*, and *Enterococcus faecium*, representing a major data source for Europe and a starting point for every AMR-related analysis [25]. An overall AMR trend in the reports is a higher AMR rate in the southern and eastern parts of Europe, especially for *E. coli* and *K. pneumoniae* [25]. Additionally, for many bacteria/antibiotic combinations, AMR rates in Balkan countries are among the highest in Europe [25].

The CAESAR network was established in 2012, while the first report on AMR data was released in 2015 with data for the year 2014. Since then, every year, more and more countries and laboratories have been involved in this network. That is the crucial part of surveillance, as it brings larger population coverage and better representativeness of the data [25]. AMR trend analysis requires high-quality data, meaning that any missing information, such as the absence of data for any year within the analysis period, fewer than 20 isolates for specific bacterial species–antimicrobial agent combinations, or low data representativeness, will prevent trend calculation [25]. A significant number of the WB countries had these issues with AMR data reporting to the CAESAR (Table 1).

**Figure 3:**
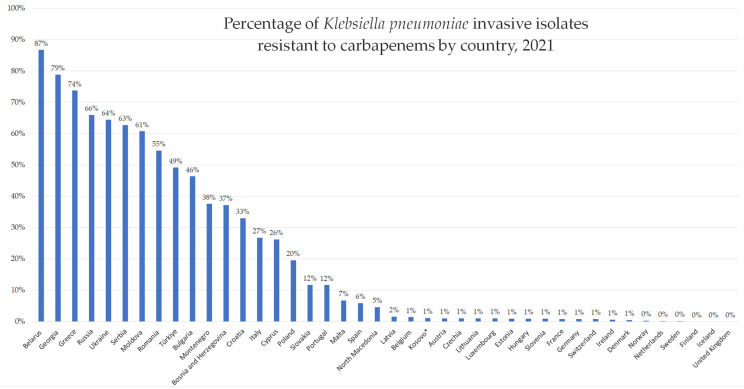
Proportion of carbapenem-resistant *Klebsiella pneumoniae* invasive isolates across European countries in 2021 [25].

When interpreting AMR data, it is important to consider indicators of data quality, such as population coverage and the representativeness of geographical areas, hospitals, and isolates [25]. For the WB countries, population coverage is overall above 70%, with the exception of Kosovo, with 59%, and Albania, for which there is no data. Geographical and hospital representativeness are high in almost all countries (excluding Albania), but the isolate representativeness is low to medium for the WB countries [25]. This particularly impacts the data regarding carbapenem-resistant *E. coli*. For *K. pneumoniae*, representativeness is better, and a continuous increase in the rate of carbapenem-resistant isolates in nearly all WB countries is apparent. Montenegro and Bosnia and Herzegovina have a significant rise in the rate of carbapenem-resistant *K. pneumoniae* (CRKP). However, it is most obvious for Serbia, which has the highest incidence of CRKP in the WBs and one of the highest in Europe (Figure 3).

Additionally, the WHO Europe Antimicrobial Medicines Consumption (AMC) network was established in 2011 with the aim to support all countries in the WHO European Region that are not EU/EEA member states in collecting and reporting data on antimicrobial consumption. Among the AMC network countries that include the entire Western Balkans, Montenegro and Serbia are the ones with the highest antimicrobial consumption, with a 27.1 and 26.6 daily defined dose/1000 inhabitants per day in 2019, respectively [26]. However, only the data of the total and relative consumption of Access, Watch, and Reserve groups of antibiotics were reported, without the data on the individual consumption of certain antibiotics (for example, carbapenems). Finally, among the WB countries, only Serbia participated in point prevalence surveys of healthcare-associated infections (HAIs) and antimicrobial use in acute care hospitals in 2016 and 2017, organized by the European Centre for Disease Prevention and Control, and the HAI prevalence for Serbia was 4.3%, while the average prevalence for participating EU/EEA countries was 5.5% [27]. However, the observed prevalence of antimicrobial use and the AMR composite index was 41.3% and 62% for Serbia and 30.5% and 31.6% for EU/EEA countries, respectively [27,28].

## 3. CRE in the Western Balkans

Considering the substantial differences in the development of national AMR surveillance systems in the WB countries [25] and the resulting data gaps, we aimed to gather more information about CRE in these countries by reviewing the available research.

The references for this review were identified through PubMed and ScienceDirect databases on 19 January 2024. The search terms used included “carbapenem resistant *Enterobacterales*”, “carbapenem resistant *Enterobacteriaceae*”, and “carbapenemase”, with each term paired with a specific country name, such as “Serbia”, “Albania”, “Bosnia and Herzegovina”, “Kosovo”, “Montenegro”, and “North Macedonia”. No limitations regarding the publication date and language were applied. This search yielded 305 results, from which duplicate entries, non-human studies, and studies unrelated to *Enterobacterales* were manually removed (*n* = 151). After a thorough review, articles lacking data on CRE/CPE or unrelated to the Western Balkans were excluded (*n* = 120). The final selection included 32 studies organized chronologically and by country.

### 3.1. Serbia

There are numerous studies from Serbia that have investigated the carbapenem resistance rate among *Enterobacterales* hospital isolates from various sources. Zec et al. analyzed carbapenem resistance in isolates from urinary tract infections (UTIs) in the Clinic for Infectious and Tropical Diseases, Clinical Center of Serbia, between 2010 and 2015 [29]. The overall resistance rates were 4.0% for imipenem and 8.5% for meropenem, with a significantly higher resistance observed in the age group of ≥65 years (15.0% and 17.1%, respectively). There was no species-related prevalence of carbapenem resistance in this study, but *E. coli* and *K. pneumoniae* represented half of all isolates. In 2013, Djuric et al. conducted a study on blood culture isolates at the Clinical Center of Serbia, the largest tertiary care facility for the adult population in the country. Their results showed imipenem and meropenem resistance in 6.5% and 3.2% of *E. coli* isolates and 67.5% and 65.1% of *K. pneumoniae* isolates, respectively [30]. Importantly, the same authors performed a similar study in the following years (2014–2016), and it showed an increasing resistance to carbapenems in *Klebsiella* spp. isolates up to 76.5%. Additionally, carbapenem resistance was detected in 66.7% *Providencia* spp. invasive isolates [31]. Another study described AMR among isolates from adult patients with hospital-acquired and ventilator-associated pneumonia in the intensive care unit (ICU) of the Clinical Center Kragujevac, Serbia, in the period of 2009–2015 [32]. Among *Enterobacterales*, isolates of *K. pneumoniae* showed the highest rate of resistance to carbapenems in the study period (50.0–62.8%), followed by *Proteus mirabilis* (26.7–40.0%) and *Enterobacter* spp. (33.3–36.4%) [32]. Popovic et al. reported an increasing trend in carbapenem resistance in *K. pneumoniae* isolates from adult ICU patients at the Clinical Center of Vojvodina, the largest tertiary care hospital in this region of Serbia, from 2014 to 2018. Meropenem resistance in *K. pneumoniae* isolates rose from 12.1% in 2014 to 57.7% in 2018 [33]. Similarly, a significant positive trend in resistance to carbapenems in *Klebsiella* spp. was confirmed in the study of Despotovic et al., in the period of 2019–2021 in adult ICU patients at the Clinic for Infectious and Tropical Diseases, Clinical Center of Serbia [34]. In the study of Gajic et al., high carbapenem resistance rates in *K. pneumoniae* isolates were found in three regional hospitals in Serbia during 2021–2022, with 62.5% resistance to imipenem and 71.3% resistance to meropenem [35]. It can be noticed that the carbapenem resistance rate varied among different hospitals, but an increase in the resistance rate during the last decade is obvious.

In addition to the aforementioned studies focusing only on resistance rates, there have been multiple studies that examined the presence of carbapenemase in CRE isolates in Serbia. The first carbapenemase-producing enterobacterial isolates were confirmed in 2008 from an outbreak in the adult surgical ICU of a university hospital in Belgrade, Serbia [36]. Those were *Proteus mirabilis* isolates with IMP (66.7%) and VIM (33.3%) MBLs. Several years later, in 2011, Mirovic et al. described the first case of the NDM-positive *K. pneumoniae* isolate, which originated from the urine of an outpatient child [37]. As Mirovic et al. suggested and as other authors described in several studies [38,39,40], NDM carbapenemase was related to the Balkan countries, and this region is considered as endemic. Evidence of the spread of resistance genes from this high-prevalence region emerged from Belgium in 2010 and from Switzerland in 2013. In a study from Belgium, two isolates from an adult female patient previously hospitalized in Serbia, *Morganella morganii* and *Enterobacter cloacae*, produced NDM carbapenemase [41]. An extensively drug-resistant *K. pneumoniae* that produced two different classes of carbapenemase, NDM-1 and OXA-48, was isolated from a 54-year-old patient in Switzerland with a history of hospitalization in the ICU in Belgrade, Serbia [42]. This isolate carried carbapenemase genes on two different plasmids, *bla*_OXA-48_ located on the IncL/M plasmid and the IncA/C plasmid that carried *bla*_NDM-1_ [42]. Multilocus sequence typing revealed that this *K. pneumoniae* belonged to ST101, a rapidly emerging pan-epidemic clone [42,43].

In the period of 2013–2014, a prospective, multinational study on carbapenemase-producing *K. pneumoniae* and *E. coli* in Europe (EuSCAPE) showed a high incidence of these isolates in Serbia [44]. In this study, carbapenemase production was confirmed by PCR in 71% of *K. pneumoniae* and 40% of *E. coli* carbapenem non-susceptible isolates [44]. The most prevalent type of carbapenemase in Serbia was NDM (49.3%), showing one of the highest rates among participating countries [44]. That is in accordance with the endemicity of NDM in the Balkans and with the results of previously mentioned studies [37,38,39,40,41,42]. Beside NDM, OXA-48 was confirmed in 13.4% and KPC in 0.5% of *K. pneumoniae* isolates from Serbia [44]. Although OXA-48 was the most common carbapenemase in *E. coli* across Europe, all *E. coli* isolates from Serbia were found to be NDM-positive [44]. In accordance with these findings, Novovic et al. detected the *bla*_NDM_ gene in an isolate of *E. coli* from a pediatric patient treated at university-affiliated tertiary care pediatric hospital in Belgrade, Serbia, in 2015 [45]. This isolate represented a novel sequence type ST5123, most similar to the clone of animal origin ST93. This finding is very significant, as it points out animals as an important reservoir of AMR. Apart from animals, environmental sources, especially water, are another major reservoir of AMR. In another study, Novovic et al. investigated the presence of beta-lactam-resistant Gram-negative bacteria in multiple water sources (rivers, lake, springheads) in Belgrade, Serbia [46]. Among carbapenem-resistant isolates, MBLs were phenotypically confirmed, but no carbapenemase genes were detected [46]. Thus, the authors questioned the real endemicity of NDM carbapenemase in Serbia, as the presence of the *bla*_NDM_ gene in the environment is considered as one of the major characteristics of endemic regions [47].

Multidrug-resistant isolates of *E. coli* and *K. pneumoniae* from the adult patients from 14 hospitals in Serbia were examined by Trudic et al. in the period of 2013–2014 to determine carbapenemase production [48]. Results showed that 45% of isolates were carbapenemase-positive, with the NDM type of enzyme confirmed in 31% of isolates, followed by OXA-48 (7.8%) and KPC (0.8%) [48]. When Novovic et al. analyzed colistin- and carbapenem-resistant isolates of *K. pneumoniae* from Serbia from the period of 2013–2016, they revealed that 85.2% of the tested isolates were OXA-48 producers [49]. Interestingly, after the research of Novovic et al. [49], there have been several studies that point to OXA-48 carbapenemase as the most frequent type in Serbia. Among them, a study by Mijac et al., analyzed CRE in premature neonates in the period of 2018–2019 [50]. In this specific population, OXA-48 was dominant (51.1%), but KPC carbapenemase was almost equally represented (47.7%). As the study was conducted in a single hospital, a high prevalence of KPC could be a result of cross-transmission in hospital wards, as a phylogenetic analysis revealed distinct clonality of *bla*_KPC_-positive *K. pneumoniae* isolates. NDM was also confirmed in a single isolate of *E. coli* [50].

**Figure 4:**
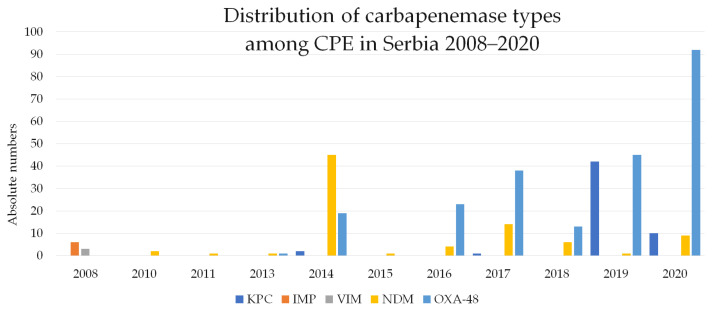
Distribution of carbapenemase types among carbapenemase-producing *Enterobacterales* (CPE) in Serbia in the period of 2008–2020.

In contrast to the studies of CRE in the hospital settings, there has been no extensive research into CRE in community settings in Serbia. Brkic et al. examined *Enterobacter* spp. community isolates from adult patients in primary healthcare settings in Belgrade, Serbia, in the period of 2016–2017 for carbapenemase production [51]. The results showed that 70.6% of carbapenemase-positive isolates carried the *bla*_NDM_ gene, which is in accordance with the global epidemiology of *Enterobacter* spp. [52]. Furthermore, 23.5% of the isolates of *Enterobacter* spp. were positive for both *bla*_NDM_ and *bla*_oxa-48_ genes. The same authors conducted a cross-sectional study of *K. pneumoniae* community isolates from 2016 to 2020 in Belgrade, Serbia, and determined OXA-48 as a predominant carbapenemase type (80.7%) [53]. In the study, 2.6% of *K. pneumoniae* isolates simultaneously harbored two types of carbapenemase genes in various combinations, namely *bla*_OXA-48-like_*/bla*_NDM_, *bla*_OXA-48-like_*/bla*_KPC_, and *bla*_KPC_*/bla*_NDM_ [53]. The co-occurrence of two, even three types of carbapenemase, is a common finding in endemic regions [10]. Changes in the predominant carbapenemase types over the years in Serbia, from MBLs in the period of 2008–2014 to OXA-48 in 2016–2020, could be observed (Figure 4).

The first study that applied whole-genome sequencing (WGS) of CRKP from Serbia, conducted by Palmieri et al., confirmed OXA-48 as the most frequent carbapenemase in the study period of 2013–2017 [54]. In this research, WGS analysis showed that the vast majority of CRKP belonged to ST101 and carried the *bla*_OXA-48_ gene on a novel plasmid p101_srb. This emphasized a great epidemiological concern, due to a high spreading potential and the high morbidity and mortality risk associated with ST101 [55]. Since ST101 has been one of the dominant STs in neighboring countries, detected in Romania, Bulgaria, and Croatia between 2010 and 2023 [56], the potential clonal dissemination of ST101 across the Balkans should be further investigated. Additional confirmation on OXA-48 spreading in Serbia was presented in the research of Cirkovic et al., who examined wastewater in Belgrade, Serbia [57]. This study evaluated clinically relevant carbapenem-resistant Gram-negative bacteria, among which the most prevalent isolates were *K. pneumoniae* (35.3%) and *Enterobacter* spp. (26.5%). All CRKP were OXA-48-positive, and half of them belonged to ST101, barring the *bla*_OXA-48_ gene on a novel p101_srb plasmid, previously detected in clinical isolates from Serbia [54]. Regarding *Enterobacter* spp., in line with the findings of Brkic et al. [49], the most common carbapenemase was NDM (44.4%) [57]. There were three *E. coli* isolates from wastewater, two with the NDM enzyme, one with OXA-48, and one *Klebsiella oxytoca* isolate with OXA-48 [57].

Several previously mentioned studies, apart from the results on carbapenem resistance, presented data on colistin resistance, albeit without an investigation of resistance mechanisms [33,35,44,48,53]. The first analysis of colistin resistance mechanisms in *K. pneumoniae* hospital isolates from Serbia from 2013 to 2016 was a study by Novovic et al. [49]. The main mechanism was the mutation of chromosomal *phoP* and *phoQ* regulatory genes. No *mcr*-1 or *mcr*-2 were detected. Palmieri et al. obtained similar results, with no detection of *mcr* genes, but with alterations in the PhoP/PhoQ regulator *mgr*B gene as a base of colistin resistance [54]. The most frequent mutation was a newly discovered substitution of the cysteine amino acid at position 28 (MgrB^C28S^) [54]. Furthermore, Cirkovic et al. confirmed the same mutation in colistin-resistant *K. pneumoniae* isolates from wastewater [57]. Additionally, a retrospective study from 2019 that analyzed the available genomes of *K. pneumoniae* isolated in 2013–2014 in Europe showed a presence of the *mcr*-9 gene in an isolate from Serbia [58]. Interestingly, Cirkovic et al. also detected the *mcr*-9 gene in a wastewater isolate of an *Enterobacter cloacae* complex [57].

### 3.2. Albania

Very few studies can be found about carbapenem resistance in Albania. Eight carbapenem-resistant isolates of *K. pneumoniae* and six of *E. coli* were included in the EuSCAPE study, but no carbapenemase producers were detected [44]. The first carbapenemase in this WB country was described in 2014 by Kostyanev et al. [59]. KPC-producing *K. pneumoniae* was isolated from a urine sample of a patient in the ICU of the University Hospital Center in Tirana, Albania, with no travel history, no previous hospitalization, or antibiotic exposure [59]. A year later, in 2015, a point-prevalence survey screening for rectal multidrug-resistant Gram-negative bacteria carriage was carried out at high-dependency wards in the country’s only tertiary care hospital in Tirana [60]. The results of phenotypic testing showed the presence of one MBL-positive isolate of *K. pneumoniae*, but no further characterization was performed. In 2018, Tafaj et al. analyzed an isolate recovered from a rectal screening sample of a patient admitted to the ICU of the University Hospital Center, Tirana, and confirmed the first NDM-positive *K. pneumoniae* in Albania [61]. The isolate was resistant to colistin due to a chromosomal mutation that affected PmrB protein, and no *mcr*1-*mcr*8 genes were detected [61]. Even though Albania is a member of a CEASAR network, the surveillance system has not yet been established in this country [61].

### 3.3. Bosnia and Herzegovina

There has been limited research on CRE in Bosnia and Herzegovina. One of the first studies was conducted in the Clinic for Gynecology and Obstetrics, University Clinical Center Tuzla, Bosnia and Herzegovina, in 2006 [62]. Resistance to imipenem was detected in 7.7% of *K. pneumoniae* isolates. Granov et al. investigated *K. pneumoniae* isolates from ICU adult patients from 2017 to 2018 in the Clinical Center, University of Sarajevo, Bosnia and Herzegovina [63]. All tested isolates were OXA-48 producers and colistin-susceptible. Two more studies investigated AMR rates in hospital isolates, but without the detection of the resistance mechanisms. Research conducted in a single hospital in Doboj, Bosnia and Herzegovina, showed a relatively low rate of carbapenem resistance in *K. pneumoniae* isolates, 2.2% were resistant to imipenem and 8.8% to meropenem, while in *E. coli* isolates, resistance to imipenem and meropenem was 0.4% and 1.4%, respectively [64]. Ljubovic et al. conducted a study in the Clinical Center, University of Sarajevo, Bosnia and Herzegovina, in the period of 2020–2021, and showed that 20% of *K. pneumoniae* isolates were resistant to carbapenems [65].

### 3.4. Kosovo

Without prejudice to positions on status and in line with UN Security Council Resolution 1244 and the International Court of Justice Opinion on the Kosovo Declaration of Independence, the data on AMR in Kosovo are represented in the CAESAR report, but with a low population coverage. Furthermore, to the best of our knowledge, there have been few studies that detected NDM-positive isolates in patients previously hospitalized in Kosovo. In April 2010, in Austria, an NDM-positive *K. pneumoniae* isolate was confirmed in a pediatric patient [66]. Bogaerts et al. reported, in 2010, an NDM carbapenemase in *K. pneumoniae* and *E. coli* isolates which originated from an adult patient previously hospitalized in Kosovo [41]. The study of Papst et al. involved a CRE isolate from Kosovo, but species and resistance mechanisms cannot be concluded from the results [67].

### 3.5. Montenegro

The first study that included CRE isolates from Montenegro in 2010 was from Bogaerts and al., who confirmed NDM carbapenemase in a Belgian adult patient who had a history of hospitalization in Montenegro. Those were two isolates, *K. pneumoniae* and *E. coli*, both carrying a *bla*_NDM_ gene [41]. In the EuSCAPE study, Montenegro belonged to the high-incidence countries regarding carbapenemase production, with all submitted *K. pneumoniae* confirmed as NDM-positive [44]. In 2013, a case of NDM-positive *K. oxytoca* was described in Poland. This isolate was from a patient with a history of hospitalization in Montenegro [68]. Mijovic et al. presented data on carbapenem resistance from 2016 to 2018 from the Institute of Public Health of Montenegro, but without an analysis of resistance mechanisms [69]. There was no carbapenem-resistant *E. coli*, but in *K. pneumoniae*, the resistance rate in the study period was 7.7% [69].

### 3.6. North Macedonia

Among three *K. pneumoniae* isolates from North Macedonia analyzed in the EuSCAPE study, two were KPC producers (66.7%) [44]. A multicenter study conducted by De Lorenzis et al., in 2019–2020, analyzed bacterial isolates from adult patients with UTIs and included 27 isolates from North Macedonia, comprising 17 *E. coli*, four KES (*Klebsiella* spp., *Enterobacter* spp., *Serratia* spp.), and six non-enterobacterial isolates [70]. Overall, the carbapenem resistance rate was 11%. However, no species-related data or resistance mechanisms were described [70]. Moser et al. presented an interesting finding in their study from 2020 about a Swiss patient in his 20s, previously hospitalized in North Macedonia because of polytrauma [71]. This patient was colonized with several CRE, among which the *bla*_NDM_-positive were *E. coli*, *K. pneumoniae*, and *Citrobacter sedlakii* isolates, as well as a *bla*_OXA-48_-positive *Providencia stuartii* strain [71].

The main findings about CPE in the WB countries are summarized in Table 2.

## 4. Conclusions and Future Directions

Despite the obvious improvement of the CAESAR data for the WB countries over the years, there are significant data gaps. The overall number of isolates per country is low to medium, which is in accordance with the paucity of studies into CRE from these countries. A limited availability of resources, such as modern equipment or training, particularly for genomic analysis, significantly contributes to the small quantity of research conducted. Serbia could be an exception and a good example, as several genomic-based studies contributed to a growing understanding of AMR in this country. The most concerning results were a detection of the new plasmid p101_srb, harboring OXA-48 carbapenemase in *K. pneumoniae* isolates from humans and wastewater samples, as well as the detection of the *mcr*-9 gene in *K. pneumoniae* and *E. cloacae* complex strains, also from different sources. These results suggest a possible interchange of resistant determinants in bacteria over time and through different niches and emphasize the need for an in-depth retrospective genomic analysis to gain a better insight into AMR spreading. While these results stem from individual projects, there is a pressing need for increased national support for microbiology laboratories.

## Figures and Tables

**Table 1:** CAESAR data on carbapenem resistance in *E. coli* and *K. pneumoniae* in the Western Balkan countries.

WB Country	2016		2017		2018		2019		2020		2021	
	EC	KP	EC	KP	EC	KP	EC	KP	EC	KP	EC	KP
Albania	-	-	-	-	-	-	-	-	-	-	-	-
Bosnia and Herzegovina	0.0	8.0	1.1	10.9	0.0	18.4	0.0	41.7	0.0	43.5	0.5	37.1
Kosovo *	NA	0.0	NA	0.0	NA	1.5	NA	0.0	NA	0.0	NA	1.1
Montenegro	0.0 ^a^	3.7 ^a^	0.0 ^a^	13.8 ^a^	0.0 ^a^	4.5 ^a^	0.0 ^a^	17.4 ^a^	0.0 ^a^	13.8 ^a^	NA	37.5
North Macedonia	0.0	12.5 ^a^	0.0	17.4 ^a^	3.7	20.5	1.2	7.3	2.0	5.1	0.0	4.6
Serbia	0.6	34.5	1.0	34.9	0.9	36.2	0.4	39.3	1.4	47.9	3.2	62.7

* This designation is without prejudice to positions on status and is in line with UN Security Council Resolution 1244 and the International Court of Justice Opinion on the Kosovo Declaration of Independence. WB: Western Balkan; EC: *Escherichia coli*; KP: *Klebsiella pneumoniae*; NA: not applicable; ^a^ a small number of isolates were tested (*n* < 30) and the percentage resistance should be interpreted with caution.

**Table 2:** Carbapenemase-producing *Enterobacterales* in the Western Balkan countries.

WB Country	Year	Species	Isolates (*n*) ^a^	Carbapenemase Type Detected (*n*, %)						Ref.
				KPC	IMP	VIM	NDM	OXA-48	Multiple Types	
Albania	2014	*K. pneumoniae*	1	1 (100)	NP	NP	NP	NP		[59]
	2018	*K. pneumoniae*	1	NP	NP	NP	1 (100)	NP		[61]
Bosnia and Herzegovina	2017–2018	*K. pneumoniae*	15	NP	NP	NP	NP	15 (100)		[63]
Kosovo *	2010	*K. pneumoniae*	1	ND	ND	ND	1 (100)	ND		[66]
	2010	*K. pneumoniae*	1	NP	NP	NP	1 (100)	NP		[41]
		*E. coli*	1	NP	NP	NP	1 (100)	NP		
Montenegro	2010	*K. pneumoniae*	1	NP	NP	NP	1 (100)	NP		[41]
		*E. coli*	1	NP	NP	NP	1 (100)	NP		
	2013	*K. oxytoca*	1	ND	NP	NP	1 (100)	NP		[68]
	2013–2014	*K. pneumoniae*	10	NP	ND	NP	10 (100)	NP		[44]
North Macedonia	2013–2014	*K. pneumoniae*	3	2 (66.7)	ND	NP	NP	NP		[44]
	2020	*K. pneumoniae*	1	NP	NP	NP	1 (100)	NP		[71]
		*E. coli*	1	NP	NP	NP	1 (100)	NP		
		*C. sedlakii*	1	NP	NP	NP	1 (100)	NP		
		*P. stuartii*	1	NP	NP	NP	NP	1 (100)		
Serbia	2008	*P. mirabilis*	9	ND	6 (66.7)	3 (33.3)	NP	ND		[36]
	2010	*M. morganii*	1	NP	NP	NP	1 (100)	NP		[41]
		*E. cloacae*	1	NP	NP	NP	1 (100)	NP		
	2011	*K. pneumoniae*	1	ND	NP	NP	1 (100)	ND		[37]
	2013	*K. pneumoniae*	1	NP	NP	NP	NA	NA	1 (100) NDM, OXA48	[42]
	2013–2014	*K. pneumoniae*	43	1 (2.4)	ND	NP	33 (76.7)	9 (20.9)		[44]
		*E. coli*	5	NP	ND	NP	5 (100)	NP		
	2013–2014	*K. pneumoniae*, *E. coli*^b^	58	1 (1.7)	NP	NP	40 (69.0)	10 (17.2)	7 (12.1) NDM, OXA48	[48]
	2015	*E. coli*	1	NP	NP	NP	1 (100)	NP		[45]
	2013–2016	*K. pneumoniae*	27	NP	NP	NP	4 (14.8)	23 (85.2)		[49]
	2013–2017	*K. pneumoniae*	45	1 (2.2)	NP	NP	2 (4.4)	37 (82.2)		[54]
	2016–2017	*Enterobacter* spp.	17	NP	NP	NP	12 (70.6)	1 (5.9)	4 (23.5) NDM, OXA48	[51]
	2018	*K. pneumoniae*	12	NP	NP	NP	NP	11 (91.7)	1 (8.3) NDM, OXA48	[57]
		*E. cloacae*	9	NP	NP	NP	4 (44.4)	NP		
		*E. coli*	3	NP	NP	NP	2 (66.7)	1 (33.3)		
		*K. oxytoca*	3	NP	NP	NP	NP	1 (33.3)		
	2018–2019	*K. pneumoniae*	87	42 (48.3)	NP	NP	NP	45 (51.7)		[50]
		*E. coli*	1	NP	NP	NP	1 (100)	NP		
	2016–2020	*K. pneumoniae*	114	10 (8.8)	NP	NP	9 (7.9)	92 (80.7)	1 (0.9) NDM, OXA48 1 (0.9) OXA-48, KPC 1 (0.9) NDM, KPC	[53]

* This designation is without prejudice to positions on status and is in line with UN Security Council Resolution 1244 and the International Court of Justice Opinion on the Kosovo Declaration of Independence. WB: Western Balkan; NP: not present; ND: not done; NA: not applicable; ^a^ total number of isolates analyzed in the study; ^b^ no species-specific results.

## Data Availability

Not applicable.
